# The Mediating Role of Acceptance Action and Self-Care in Diabetes Self-Stigma’s Impact on Type 2 Diabetes Quality of Life: A Cross-Sectional Study

**DOI:** 10.3390/bs13120993

**Published:** 2023-12-01

**Authors:** Kawoun Seo

**Affiliations:** Department of Nursing, Joongbu University, Chungnam 32713, Republic of Korea; kwseo@joongbu.ac.kr

**Keywords:** acceptance and commitment therapy, diabetes mellitus, self-care, mediating effect, social stigma, quality of life

## Abstract

Recently, the quality of life of individuals with diabetes has been reduced, owing to self-stigma that occurs in the process of managing the disease. This process can be improved by accepting diabetes. This study aimed to verify the dual mediating effect of acceptance action and diabetes self-care on the effect of diabetes self-stigma on the quality of life of individuals with type 2 diabetes mellitus (T2DM) in Korea. In this study, 300 of 400 data collected to develop and evaluate health equilibrium tools for individuals with T2DM were randomly selected and analyzed. Data were collected from 1 September 2020 to 31 September 2020 using a structured online questionnaire. For data analysis, descriptive statistics and Pearson’s correlation analysis were performed using the Statistical Package for the Social Sciences (SPSS), version 24.0. Additionally, the dual mediation effect was analyzed using PROCESS Macro for SPSS, version 4.1. Acceptance action (B = −0.088, 95% confidence interval [CI], −0.127 to −0.054) and diabetes self-care (B = 0.046, 95% CI, 0.022–0.072) had a mediating effect on the relationship between diabetes self-stigma and quality of life in patients with T2DM in Korea. In particular, these two variables had dual mediating effects (B = 0.017, 95% CI, 0.015–0.019). This study confirmed that diabetes self-care and quality of life can be increased by improving acceptance behavior to overcome the negative impact of self-stigma on the quality of life of patients with T2DM. Establishing a strategy to increase acceptance action as part of an intervention to reduce the negative impact of self-stigma on the quality of life of patients with T2DM is necessary.

## 1. Introduction

Diabetes mellitus (DM) has three main types: type 1 DM (T1DM), type 2 DM (T2DM), and gestational DM. While T1DM results from a complete lack of insulin production in the body, and gestational DM occurs during pregnancy due to insulin resistance caused by hormonal changes, T2DM is primarily characterized by the ineffective use of produced insulin, and its prevalence is increasing worldwide [1]. In Korea, the prevalence is so high that one in seven adults aged > 30 years is a T2DM patient [2]. Individuals with DM experience a decline in both their overall quality of life and disease-related quality of life compared to the general population. This decline is attributed to the ongoing self-management requirements and the stress associated with the condition [3,4,5,6]. According to the World Health Organization (WHO), quality of life encompasses an individual’s perception of their position in life relative to their goals, expectations, standards, and interests, considering the cultural and value system they belong to [7]. Therefore, improving quality of life becomes crucial for patients with DM because it serves as a comprehensive assessment of their overall well-being while managing the disease, emphasizing the need for interventions aimed at enhancing their quality of life.

Lifestyle management, including diet control, exercise, sobriety, and smoking cessation, plays a crucial role in managing DM alongside medication treatment [1,8]. However, patients with DM often experience constant judgment and scrutiny from others during their management journey [9]. This may be because they believe that diabetes is a disease caused by poor health care. Alternatively, they may be worried that if people find out they have diabetes, others will criticize them for not adhering to a diabetes diet [10]. Thus, they develop a negative self-perception referred to as diabetes self-stigma. This self-stigma causes individuals with DM to devalue themselves and experience negative emotions [11]. Research indicates that self-stigma related to the disease reduces the quality of life of individuals with DM by diminishing self-esteem and self-efficacy [12]. Moreover, studies have demonstrated that self-stigma in patients with DM contributes to a decline in their overall quality of life [8].

Self-stigma arises when individuals fail to acknowledge their disease and maintain a healthy self-image after being diagnosed with a chronic illness [8,13]. Studies have shown that individuals with DM struggle to establish a positive self-perception that balances their physical condition and social responsibilities [14,15]. This self-stigma not only diminishes the quality of life of individuals with DM but also hampers their self-care efforts, such as the inability to manage their diet when eating with others [8,14]. One reason for this is that patients with DM prefer not to disclose their condition to others, leading them to conceal their dietary management [11,16]. Consequently, their reduced self-care behaviors further compromise their quality of life, intensify the psychological burden of unmanaged illness, and increase anxiety about potential complications [8,10,17]. To summarize, self-stigmatization among individuals with DM negatively impacts their self-care practices and, subsequently, their overall quality of life. However, accepting one’s disease can improve this process.

Acceptance action encompasses the willingness of individuals to embrace their thoughts and emotions while aligning their actions with their values and goals [18]. A qualitative investigation focusing on the experiences of patients with DM revealed that these individuals thrived by accepting their condition as an integral part of their lives and adjusting various aspects to effectively manage DM [10]. Notably, research has demonstrated that acceptance action serves as a mediating factor between self-stigma and quality of life among patients with DM [8]. Furthermore, studies have validated the effectiveness of acceptance and commitment interventions in reducing stress levels and enhancing treatment adherence among patients with DM [19,20]. Despite these notable advancements, the existing literature on the influence of self-stigma on the quality of life of patients with DM lacks a comprehensive exploration of the pathways involving acceptance action and self-care.

Therefore, this study aimed to investigate the mediating effects of acceptance action and self-care on the correlation between self-stigma and quality of life among individuals with T2DM. Additionally, this study aimed to identify the dual mediating effect of acceptance action and self-care on the relationship between self-stigma and quality of life. The primary objective of this study is to furnish fundamental data for developing an intervention program aimed at enhancing the quality of life of patients with T2DM.

## 2. Materials and Methods

### 2.1. Study Design

This cross-sectional study aimed to explore the interconnected roles of acceptance action and self-care as mediators in the relationship between self-stigma and quality of life among individuals diagnosed with T2DM.

### 2.2. Data Source and Participants

This study is a secondary analysis study conducted by randomly extracting data from 300 of 400 patients with DM to conduct a study on the “development and evaluation of health equilibrium in diabetes patients (JIRB-2020101201-02-201101)”. To gather data for the initial study, a survey was administered to individuals who had been diagnosed with DM by a medical professional using a survey organization (PMI). The survey had specific criteria for inclusion, which were as follows: (1) participants must be adults aged ≥19 years, (2) individuals who can self-manage their DM, (3) respondents who had a sufficient understanding of the questionnaire’s content and could complete it online, and (4) individuals who voluntarily agreed to participate in the survey and successfully completed it. The exclusion criteria for participants were (1) those aged <18 years, (2) those who had not been diagnosed with diabetes by an endocrinologist, and (3) those who were unable to manage DM on their own because of physical or mental disabilities. A sample size of 225 individuals was required to validate the study’s objectives, representing 15 times the number of parameters as per the criteria outlined by Hair et al. [21]. Nevertheless, to enhance the precision of the statistical outcomes, the analysis was conducted with a sample size of 300. Out of the 400 data entries obtained through this process, 300 patients with type 2 diabetes were selected as a random sample using the Statistical Package for the Social Sciences (SPSS) program. Patients who did not complete the survey were not included in the study population; therefore, there was no missing data among the 300 data entries. Before proceeding with this study, it was exempted from deliberation by the Institutional Review Board of Joongbu University (JIRB-2023070902-01-230714).

### 2.3. Measures

The survey included 7 general characteristics, 16 questions on diabetes self-stigma, 26 questions on quality of life, 16 questions on acceptance action, and 11 questions on diabetes self-care. General characteristics consisted of age, sex, education level, having a spouse, duration of illness, treatment type, and experience of diabetes education. Diabetes education includes education on all diseases related to diabetes, as well as education on self-care methods, depending on whether or not the person participated in diabetes-related education.

#### 2.3.1. Diabetes Self-Stigma Scale

The Diabetes Self-Stigma Scale, developed by Seo and Song [11], was used to assess self-stigma associated with DM. This scale comprises 16 items categorized into the following four subdomains: comprehensive inability, social withdrawal, self-devaluation, and apprehensive feeling. Each item is rated on a 5-point Likert scale ranging from 1 (“strongly disagree”) to 5 (“strongly agree”), and in this study, all scores were inversely transformed to calculate the average. Therefore, the higher the scores, the lower the levels of self-stigma. This tool had a Cronbach’s α of 0.89 during development [11]. The reliability of this tool in this study was Cronbach’s α = 0.93.

#### 2.3.2. Quality of Life Scale

Min et al. [22] translated and assessed the validity of the Korean version of the WHO Quality of Life-BREF (WHOQOL-BREF), originally developed by the WHO [23], as a measure of quality of life. The scale comprises 26 questions, categorized into the following four areas: physical health (7 items), psychological well-being (6 items), social interactions (3 items), living environment (8 items), and overall quality of life (2 items). Each question is rated on a 5-point Likert scale, with higher scores indicating a higher quality of life. Notably, negative questions (Nos. 3, 4, and 26) were reverse-coded. In Min et al. ’s (2000) study [22], the scale demonstrated a Cronbach’s α coefficient of 0.89, whereas this study observed a reliability coefficient of Cronbach’s alpha = 0.92.

#### 2.3.3. Acceptance Action Scale

For acceptance action, Moon [24] translated the acceptance and action questionnaire developed by Hayes et al. [18]. This tool consists of 16 items evaluated on a 7-point Likert scale ranging from 1 point for “not at all” to 7 points for “always”. A higher total score indicates a higher acceptance behavior. In Moon’s study [24], the scale demonstrated a Cronbach’s α coefficient of 0.82, whereas in the present study, a Cronbach’s α coefficient of 0.82 was observed.

#### 2.3.4. Diabetes Self-Care Scale

For diabetes self-care, the Summary of Diabetes Self-Care Activities Questionnaire (SDSCA), developed by Toobert and Glasgow and modified by Toobert, Hampson, and Glasgow [25], was used. The SDSCA was translated into Korean, which was then validated by Chang and Song [26]. This tool consists of 11 questions, categorized into the following five subdomains: diet, exercise, blood sugar test, foot care, and smoking. In a study that verified the validity and reliability of the Korean version of the SDSCA [26], the Cronbach’s α was 0.77, whereas in the present study, the Cronbach’s α was estimated to be 0.81.

### 2.4. Statistical Analysis

The statistical analyses were performed using SPSS (version 24.0; IBM Corp, Armonk, NY, USA) and PROCESS Macro for SPSS (version 4.1) developed by Hayes and Rockwood [27]. Descriptive statistics were employed to analyze the sample characteristics, encompassing the study variables. Pearson’s correlation coefficient was used to ascertain the correlations among diabetes self-stigma, acceptance action, diabetes self-care, and quality of life. Model 6 of the PROCESS Macro for SPSS version 4.1 (IBM Corp, Armonk, NY, USA) was used to examine the dual mediating effect of acceptance action and diabetes self-care on the relationship between diabetes self-stigma and quality of life. Significance of the results was tested with a 95% confidence interval at a significance level of 0.05.

## 3. Results

### 3.1. General Characteristics

The ages of the participants ranged from 22 to 80 years, with an average age of 54.40 years. Most participants were males, accounting for 82.0% of the total study sample. Among the sample, 71.3% had attained a university education or higher, and 77.0% were married or had a spouse. The average duration of DM among the participants was 7.95 years, ranging from 1 to 41 years. Note that 81% of the participants were taking oral medications, and 38.3% of them received DM-related education.

The average score for diabetes self-stigma was 3.44 ± 0.70, and that for acceptance action was 4.17 ± 0.40. The average diabetes self-care score was 3.09 ± 1.26 points, and the average quality of life score was 3.25 ± 0.45 points (Table 1).

### 3.2. Correlations between Study Variables

Quality of life was negatively correlated with diabetes self-stigma and positively correlated with acceptance action and diabetes self-care. In other words, the higher the diabetes self-stigma, the lower the quality of life, and the higher the acceptance action and self-care, the higher the quality of life. Diabetes self-stigma was negatively correlated with acceptance action and positively correlated with diabetes self-care. In other words, the higher the diabetes self-stigma, the lower the acceptance action but the higher the self-care. No significant correlation was noted between acceptance behavior and diabetes self-care (Table 2).

### 3.3. Dual Mediating Effect of Acceptance Action and Diabetes Self-Care on the Relationship between Diabetes Self-Stigma and Quality of Life

**Figure 1:**
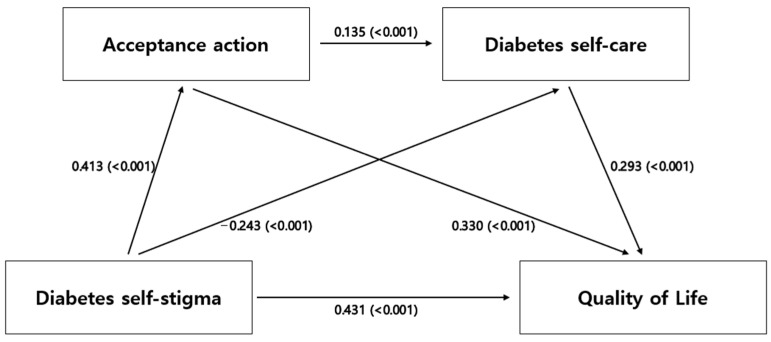
Mediating effect of variables.

Table 3 and Figure 1 show the results of analyzing the mediating effects of acceptance action and diabetes self-care on the relationship between diabetes self-stigma and quality of life. In the first step, the impact of diabetes self-stigma (independent variable) on quality of life (dependent variable) was confirmed. The results were found to be significant, with an explanatory power of 18.5%. In the second step, the effect of diabetes self-stigma (independent variable) on acceptance action (parameter 1) was confirmed. The result was also found to be significant, with an explanatory power of 17.0%. In the third step, the effects of diabetes self-stigma (independent variable) and acceptance action (mediating variable 1) on diabetes self-care (mediating variable 2) were confirmed. This result was also found to be significant, with an explanatory power of 5.0%. Finally, in step 4, the effects of diabetes self-stigma (independent variable), acceptance action (mediating variable 1), and diabetes self-care (mediating variable 2) on quality of life (dependent variable) were confirmed, and this result was also found to be significant, with an explanatory power of 38.0%.

Table 4 illustrates the comparison of the differences in effect values for each path to comprehend the significance and magnitude of the dual mediation effect. The mediating effect (indirect 1) of diabetes self-stigma through acceptance action on quality of life was statistically significant. This implies that diabetes self-stigma impacts quality of life through acceptance action. Diabetes self-care (indirect 2) was also statistically significant. This indicates that diabetes self-stigma affects quality of life through diabetes self-care. Lastly, the mediating effect of diabetes self-stigma on quality of life through the dual mediation of acceptance action and diabetes self-care (indirect 3) was also statistically significant. This means that diabetes self-stigma influences self-care through acceptance action, and altered self-care affects the quality of life of individuals with diabetes. When comparing the size of each effect on the impact of diabetes self-stigma on quality of life, the mediating effect of acceptance action was found to be greater than that of diabetes self-care. Additionally, the dual mediating effect of acceptance action and diabetes self-care was greater than the mediating effect of diabetes self-care alone. Moreover, the mediation of acceptance action alone was found to be more effective than the dual mediation of acceptance action and diabetes self-care. Therefore, the mediating effect of acceptance action was confirmed to be the strongest in terms of how diabetes self-stigma influences quality of life.

## 4. Discussion

The primary objective of this study was to validate the significance of acceptance action and self-care in influencing the impact of self-stigma on the quality of life of individuals with T2DM. The findings revealed that both acceptance action and self-care act as mediators in the relationship between self-stigma and quality of life. Notably, the study highlighted that these factors have dual mediating effects.

The participants in this study had an average age distribution of 54.4 years. There were more men (82.0%) than women, and 71.3% of them had university or higher education, which was higher than that reported in other diabetes-related studies conducted in Korea [11,17]. This may be because the survey in this study was conducted online. The average disease duration of the participants in this study was 7.95 years, ranging from a minimum of 1 year to a maximum of 41 years. This was a short period compared with the average disease duration of 10.43 years for participants analyzed in Korea using the National Health Insurance Service health examination data [28]. The duration of diabetes is related to chronic diabetes complications; therefore, it is a known fact that the longer the disease period, the more diabetes patients pay attention to health management [28,29]. Consequently, to generalize the results of this study in the future, there is a need to analyze the effect size according to the duration of diabetes.

The first hypothesis of this study was supported: acceptance action was found to play a mediating role in the relationship between self-stigma and quality of life among patients with T2DM. Furthermore, this study found a positive correlation between self-stigma, acceptance action, and quality of life. Specifically, acceptance action was found to mediate the correlation between self-stigma and quality of life. This means that when patients with T2DM had a less negative self-perception of their condition, their acceptance action increased, improving their quality of life. These results agree with those of Seo [8]. Diabetes self-stigma often arises when individuals struggle to come to terms with their DM diagnosis, as noted by Larsen [13]. Qualitative research on the experiences of patients with DM indicated that many initially find accepting their condition upon diagnosis challenging [10]. However, as time goes on, most individuals learn to embrace DM as a part of themselves, enhancing their quality of life [10]. Another study on individuals with DM revealed that accepting and living with the condition had positive effects [30]. Consequently, those with higher levels of self-stigma regarding DM can enhance their quality of life by improving their acceptance action. To improve acceptance action among patients with DM in the future and validate its effectiveness, researching and developing a diabetes acceptance and commitment treatment program is essential. This program could prove beneficial in supporting patients with DM in coming to terms with their condition and improving their quality of life.

This study found that self-care among patients with T2DM played a significant role in mediating the relationship between diabetes self-stigma and quality of life, supporting the second hypothesis. The study revealed that diabetes self-stigma was negatively correlated with self-care, whereas self-care was positively correlated with quality of life. This means that higher levels of diabetes self-stigma improved self-care among patients with T2DM, and that improved self-care subsequently enhanced the quality of life of these patients. Interestingly, our findings differed from those of previous studies, as the increase in self-stigma was associated with an increase in self-care, improving quality of life. In contrast, earlier studies suggested that self-stigma negatively affects self-care. For instance, Wang et al. [3] observed that high self-stigma directly influenced a reduction in quality of life but indirectly improved hemoglobin A1C levels. In the study by Seo and Song [11], some individuals with diabetes self-stigma, particularly those experiencing social withdrawal, were diligent in self-care despite the challenges posed by dietary restrictions in certain cultural settings [11]. Although some studies, such as those by Cho et al. [31] and Mahdilouy and Ziaeirad [32], indicated that self-stigma is positively correlated with self-care, the direction of the relationship remained less clear. Another point to note is that social activities were limited due to COVID-19 during the survey period for the present study. Because diabetes patients’ self-stigma is considerably influenced by social activities, the relationship with self-care may have been derived more clearly than in previous studies. Overall, qualitative research is required to delve deeper into the self-stigma experiences of patients with T2DM and explore how self-stigma influences diabetes self-care through specific pathways. Such investigations can offer valuable insights into improving the overall management of DM and enhancing the well-being of those living with the condition.

The findings of this study demonstrate that acceptance action and self-care serve as dual mediators in the relationship between diabetes self-stigma and quality of life. The third hypothesis was also supported, revealing that lower self-stigma leads to higher acceptance action, which, in turn, enhances self-care and improves the quality of life of individuals with T2DM. Notably, an acceptance and commitment treatment program applied to Korean adolescents with T1DM reduced stress and improved self-care and quality of life [33]. The findings of hypothesis 2 are particularly interesting: in the absence of acceptance action, higher self-stigma led to better self-care and subsequently improved quality of life. However, introducing acceptance action altered this relationship, showing that reduced self-stigma was correlated with improved acceptance action, enhancing self-care. Addressing self-stigma in patients with DM is crucial because it negatively impacts self-efficacy and self-esteem and contributes to various psychological problems. Effectively managing self-stigma becomes essential for the psychological well-being and overall quality of life of these individuals. Based on the results of this study, patients with T2DM and high self-stigma can improve self-care and enhance their quality of life by fostering acceptance action. Therefore, it is necessary to develop programs to improve acceptance action as an intervention program to enhance the quality of life of type 2 diabetes patients with high self-stigma. Additionally, as improvement in self-care can be anticipated through this program, further research is needed to develop and evaluate a self-care intervention program by addressing self-stigma and enhancing acceptance action in type 2 diabetes patients.

Nevertheless, this study has several limitations. First, caution is needed in broadly applying our findings, as the number of participants is relatively small compared to the number of individuals with DM in Korea. Second, the online survey method limits participation to individuals with access to a mobile phone or PC, and the survey consists primarily of men. Therefore, generalization of the results requires careful consideration. Third, relying on a parallel research design limits the ability to establish causal relationships between study variables. Despite these limitations, this study may provide valuable insights to guide intervention studies assessing changes in relevant variables following the implementation of acceptance action programs for individuals with T2DM.

## 5. Conclusions

This study aimed to examine the impact of acceptance action and self-care on the relationship between diabetes self-stigma and quality of life in individuals with T2DM. The findings revealed that acceptance action and self-care played a significant dual mediating role in the relationship between diabetes self-stigma and quality of life in individuals with T2DM. These fundamental data offer valuable practical recommendations emphasizing the importance of acceptance action in enhancing self-care and overall quality of life of individuals experiencing diabetes self-stigma. To enhance the self-care and quality of life of individuals with T2DM in the future, assessing the level of self-stigma and acceptance action and developing tailored acceptance programs accordingly, considering the varying degrees of self-stigma and acceptance action among participants, are crucial.

## Figures and Tables

**Table 1:** General characteristics and independent variables of the participants (N = 300).

Characteristics	Categories	*n* (%) or M (SD)	Range Min–Max
Age		54.40 (10.51)	22–80
Sex	Male	246 (82.0)	
	Female	54 (180)	
Education level	≤Middle school	11 (3.7)	
	High school	75 (25.0)	
	≥University	214 (71.3)	
Having a spouse	Yes	231 (77.0)	
	No	69 (23.0)	
Duration of illness		7.95 (7.05)	1.00–41.00
Therapy type	Oral medication	243 (81.0)	
	Insulin	21 (7.0)	
	Oral medication + insulin	36 (12.0)	
Experience of diabetes education	Yes	115 (38.3)	
	No	185 (61.7)	
Diabetes self-stigma		3.44 (0.70)	1.13–5.00
Acceptance action		4.17 (0.40)	2.50–5.94
Diabetes self-care		3.09 (1.26)	0.10–6.50
Quality of life		3.25 (0.45)	2.08–4.81

M = Mean; SD = Standard Deviation; Min = minimum; Max = maximum.

**Table 2:** Correlations among study variables (N = 300).

Variables	Diabetes Self-Stigma	Acceptance Action	Diabetes Self-Care
	r (*p*)	r (*p*)	r (*p*)
Acceptance action	−0.41 (<0.001)	1	
Diabetes self-care	0.18 (<0.001)	0.03 (0.546)	1
Quality of life	−0.43 (<0.001)	0.48 (<0.001)	0.24 (<0.001)

**Table 3:** Dual mediating effect of acceptance action and diabetes self-care on the relationship between diabetes self-stigma and quality of life (N = 300).

No	Variables	β (Coeffect)	SE	*p*	95% CI		Ajd. R^2^
					LLCI	ULCI	
1	DSS→QoL	0.431	0.033	<0.001	0.212	0.345	0.185
2	DSS→AA	0.413	0.030	<0.001	0.176	0.294	0.170
3	DSS→DSC	−0.243	0.110	<0.001	−0.651	−0.215	0.050
	AA→DSC	0.135	0.194	<0.001	0.041	0.805	
4	DSS→QoL	0.349	0.033	<0.001	0.160	0.291	0.380
	AA→ QoL	0.330	0.057	<0.001	0.261	0.487	
	DSC→ QoL	0.293	0.017	<0.001	0.072	0.139	

SE = Standard Error; CI = confidence interval; LLCI = Lower Limit of Confidence Interval; ULCI = Upper Limit of Confidence Interval; Adj. = Adjusted; DSS = diabetes self-stigma; QoL = quality of life; AA = acceptance action; DSC = diabetes self-care.

**Table 4:** Validation of mediating effect (bootstrapping) (N = 300).

Variables		Effect	Boot SE	95% CI	
				LLCI	ULCI
Indirect 1	DSS→AA→QoL	0.136	0.026	0.086	0.191
Indirect 2	DSS→DSC→QoL	−0.071	0.020	−0.112	−0.034
Indirect 3	DSS→AA→DSC→QoL	0.016	0.008	0.000	0.034
Differences (ΔB)	Indirect 1–Indirect 2	0.207	0.031	0.146	0.270
	Indirect 1–Indirect 3	0.120	0.027	0.070	0.175
	Indirect 2–Indirect 3	−0.087	0.024	−0.138	−0.044

LLCI = Lower Limit of Confidence Interval; ULCI = Upper Limit of Confidence Interval; DSS = diabetes self-stigma; QoL = quality of life; AA = acceptance action; DSC = diabetes self-care; SE = Standard Error.

## Data Availability

The data presented in this study are available on request from the corresponding author.
